# Carvacrol Codrugs: A New Approach in the Antimicrobial Plan

**DOI:** 10.1371/journal.pone.0120937

**Published:** 2015-04-10

**Authors:** Ivana Cacciatore, Mara Di Giulio, Erika Fornasari, Antonio Di Stefano, Laura Serafina Cerasa, Lisa Marinelli, Hasan Turkez, Emanuela Di Campli, Soraya Di Bartolomeo, Iole Robuffo, Luigina Cellini

**Affiliations:** 1 Department of Pharmacy, University “G. d’Annunzio” Chieti-Pescara, Chieti, Italy; 2 Department of Molecular Biology and Genetics, Faculty of Science, Erzurum Technical University, Erzurum, Turkey; 3 Institute of Molecular Genetics, National Research Council (CNR) section of Chieti, Chieti, Italy; Animal Health and Veterinary Laboratories Agency, UNITED KINGDOM

## Abstract

**Objective:**

The increasing prevalence of antibiotic-resistant bacterial infections led to identify alternative strategies for a novel therapeutic approach. In this study, we synthesized ten carvacrol codrugs – obtained linking the carvacrol hydroxyl group to the carboxyl moiety of sulphur-containing amino acids via an ester bond – to develop novel compounds with improved antimicrobial and antibiofilm activities and reduced toxicity respect to carvacrol alone.

**Method:**

All carvacrol codrugs were screened against a representative panel of Gram positive (*S*. *aureus* and *S*. *epidermidis*), Gram negative (*E*. *coli* and *P*. *aeruginosa*) bacterial strains and *C*. *albican*s, using broth microdilution assays.

**Findings:**

Results showed that carvacrol codrug **4** possesses the most notable enhancement in the anti-bacterial activity displaying MIC and MBC values equal to 2.5 mg/mL for all bacterial strains, except for *P*. *aeruginosa* ATCC 9027 (MIC and MBC values equal to 5 mg/mL and 10 mg/mL, respectively). All carvacrol codrugs **1-10** revealed good antifungal activity against *C*. *albicans* ATCC 10231. The cytotoxicity assay showed that the novel carvacrol codrugs did not produce human blood hemolysis at their MIC values except for codrugs **8** and **9**. In particular, deepened experiments performed on carvacrol codrug **4** showed an interesting antimicrobial effect on the mature biofilm produced by *E*. *coli* ATCC 8739, respect to the carvacrol alone. The antimicrobial effects of carvacrol codrug **4** were also analyzed by TEM evidencing morphological modifications in *S*. *aureus*, *E*. *coli*, and *C*. *albicans*.

**Conclusion:**

The current study presents an insight into the use of codrug strategy for developing carvacrol derivatives with antibacterial and antibiofilm potentials, and reduced cytotoxicity.

## Introduction

The recent increase of bacterial resistance/tolerance to antimicrobial agents—due to genotypical bacterial modifications—encourages the scientific community towards the research of novel therapeutic approaches to treat microbial infections [1–5].

The antibacterial properties of natural products, such as essential oils and their components, are widely explored by both industrial and academic fields [6–7]. Notably, essential oils are known for their antibacterial, antifungal, and insecticidal activities. Data reported that Gram positive bacteria are more susceptible to the phenolic components of essential oils than Gram negative bacteria since they act as membrane permeabilisers [8].

Carvacrol (2-methyl-5-[1-methylethyl]phenol), a phenolic monoterpenoid, is a constituent of essential oils produced by numerous aromatic plants and spices [9]. To date, several reports indicate that this monoterpenoid exhibits antimicrobial, fungicidal, anticarcinogenic, and antitumor activities [10–13]. Regarding its antimicrobial activity, carvacrol causes destabilization of bacterial membrane, decrease in the membrane potential, dissipation of pH gradients, and perturbation of lipid fractions of bacterial cytoplasmatic membranes [14, 15]. The presence of the hydroxyl group and a delocalized electron system is the structural requirement for the antibacterial activity of carvacrol [16].

Currently, some carvacrol analogs are reported in literature [17] but no example of carvacrol codrug is described. The codrug approach is commonly used to improve physicochemical, biopharmaceutical, and drug delivery properties of therapeutic agents [18–20]. In direct-coupled codrugs both drugs are directly conjugated to each other via an ester/amide bond that, after enzymatic cleavage, is able to release both drugs individually. This approach has been successfully employed to synthesize anti-Alzheimer, anti-Parkinson, anticancer, antiviral, and antibacterial codrugs [21–24].

**Fig 1:**
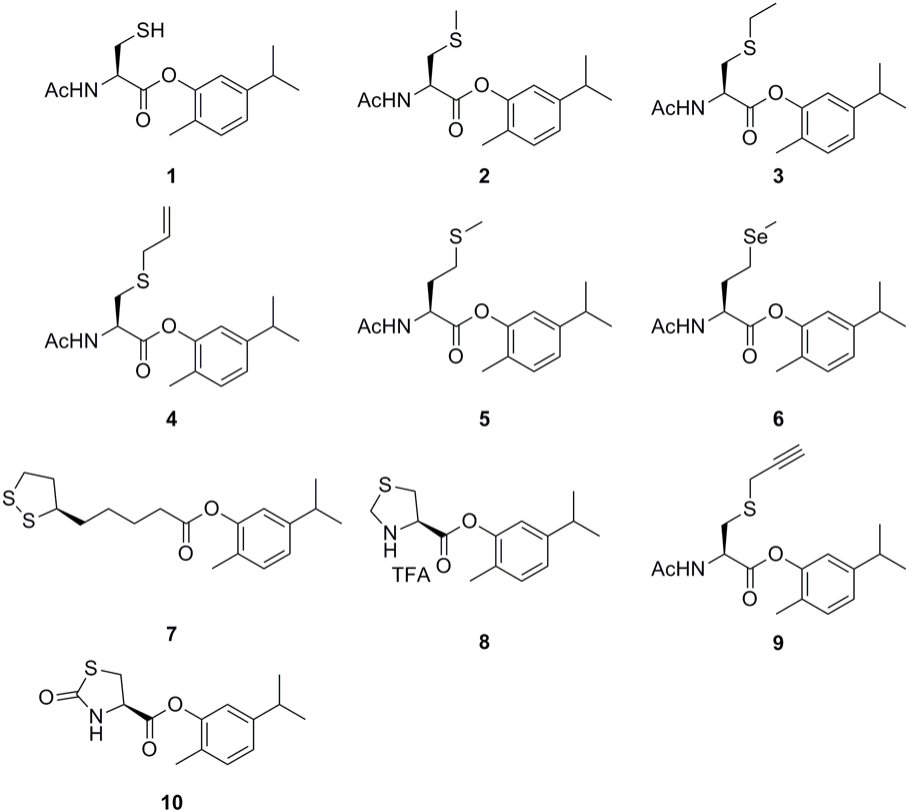
Chemical structures of carvacrol codrugs 1–10.

In the present study, the direct-coupled codrug strategy was used for generating new and more effective antimicrobial carvacrol codrugs with antibiofilm properties. Hence, we report the synthesis, antimicrobial, and antibiofilm activities of carvacrol codrugs **1–10** obtained by linking the carvacrol hydroxyl group to the carboxyl moiety of sulphur-containing amino acids via an ester bond (Fig 1). This approach allows us to obtain carvacrol codrugs endowed with antimicrobial and antibiofilm properties and, at the same time, minimize the potential toxic effects of carvacrol [25] by chemically masking the free phenolic group through linkage to sulphur derivatives [N-Acetyl-cysteine (NAC) (codrug **1**), several alkyl cysteine analogs (codrugs **2–4**, **9**), methionine (codrug **5**), selenomethionine (codrug **6**), (R)-α-lipoic acid (codrug **7**), and cyclic cysteine derivatives (codrugs **8**, **10**)].

The antibacterial and antibiofilm activities of some sulphur-containing amino acids are broadly discussed in literature. The well-known anti-biofilm properties of NAC—a mucolytic agent with antioxidant properties—could improve the antimicrobial profile of carvacrol. In fact, NAC is able to influence the formation of biofilm by *Staphylococcus epidermidis*, inhibit biofilms produced by *Pseudomonas aeruginosa*, and reduce the damage caused during *Helicobacter pylori* infections [26–28]. Moreover, sulphurated compounds extracted from garlic, such as allyl-cysteine, have been shown to have antibacterial, antifungal, antiviral, and antiprotoazoal activities [29]. Also, as demonstrated by Fujisawa *et al*. [30], sulphurated compounds containing the S-allyl moiety (such as allicin) might be an offensive tool against bacteria. Furthermore, some sulphur-containing aminoacids, such as methionine, are able to neutralize toxins produced by *Candida albicans*, such as the gliotoxin which causes extensive damage to the immune system by killing various kinds of white blood cells [31]. Starting from these considerations, the effect of all carvacrol codrugs **1–10** containing sulphur moieties was assayed against Gram positive, Gram negative, and *C*. *albicans* reference microorganisms.

## Materials and Methods

### Chemistry

Carvacrol, compounds **11–15**, **22**, **24**–**26**, and **28** were purchased from Sigma Chemical Co. (St Louis Mo, USA). All other chemicals used were of the highest purity commercially available.

^1^H- and ^13^C-NMR spectra were recorded on a Varian VXR 300-MHz spectrometer. Chemical shifts are reported in parts per million (δ) downfield from the internal standard tetramethylsilane (Me_4_Si). The LC-MS/MS system used consisted of an LCQ (Thermo Finnigan) ion trap mass spectrometer (San Jose, CA) equipped with an electrospray ionization (ESI) source. The capillary temperature was set at 300°C and the spray voltage at 4.25 kV. The fluid was nebulized by use of nitrogen (N_2_) as both the sheath gas and the auxiliary gas. The identity of all new compounds was confirmed by NMR data and LC-MS/MS system; homogeneity was confirmed by thin-layer chromatography (TLC) on Merck 60 F_254_ silica gel. Solutions were routinely dried over anhydrous sodium sulfate prior to evaporation. Chromatographic purifications were performed on a Merck 60 70–230 mesh ASTM silica gel column.

#### General method for acetylation

Acetic anhydride (6.6 mmol) was added to a stirred solution of alkylated cysteine derivatives (5.5 mmol) in acetic acid (11.1 mL) and the reaction mixture was left under stirring at room temperature for 1 h. The solvent was removed under vacuum giving the corresponding *N*-acetylated aminoacids which were used without further purification.

#### N-Ac-Cys(Trt)-OH (16)

Yield: 59%; R_*f*_ = 0.11, CH_2_Cl_2_:MeOH (9:1); ^1^H-NMR (300 MHz, 298.2 K, DMSO-d_6_): δ = 1.80 (s, 3H, Ac), 2.32–2.46 (m, 2H, Cys β-CH2), 4.05–4.18 (m, 1H, Cys α-CH), 7.20–7.34 (m, 15H, Ar), 8.22–8.24 (d, 1H, Cys NH); ^13^C-NMR (300 MHz, 298.2 K, DMSO-d_6_): δ = 23.3 (Ac), 33.91 (Cys β-CH2), 52.34 (Cys α-CH), 66.43 (C Trt), 126.12–143.98 (Ar), 178.6 and 178.8 (2 x CO).

#### N-Ac-Cys(Methyl)-OH (17)

Yield: 99%; R_*f*_ = 0.11, AcOEt:MeOH (9:1); ^1^H-NMR (300 MHz, 298.2 K, DMSO-d_6_): δ = 1.83 (s, 3H, Ac), 2.04 (s, 3H, Cys S-CH_3_), 2.66–2.84 (m, 2H, Cys β-CH2), 4.36–4.40 (m, 1H, Cys α-CH), 8.21–8.24 (d, 1H, Cys NH); ^13^C-NMR (300 MHz, 298.2 K, DMSO-d_6_): δ = 15.88 (Cys S-CH_3_), 23.04 (Ac), 35.74 (Cys β-CH2), 52.20 (Cys α-CH), 173.05 and 178.31 (2 x CO).

#### N-Ac-Cys(Ethyl)-OH (18)

Yield: 97%; R_*f*_ = 0.24, CH_2_Cl_2_:MeOH (9:1); ^1^H-NMR (300 MHz, 298.2 K, DMSO-d_6_): δ = 1.11–1.19 (t, 3H, Cys S-CH_2_C*H*_*3*_), 1.82 (s, 3H, Ac), 2.51–2.55 (q, 2H, Cys S-C*H*_*2*_CH_3_), 2.67–2.90 (m, 2H, Cys β-CH2), 4.32–4.36 (m, 1H, Cys α-CH), 8.22 (d, 1H, Cys NH); ^13^C-NMR (300 MHz, 298.2 K, DMSO-d_6_): δ = 15.22 (Cys S-CH_2_ *C*H_3_), 23.02 (Ac), 26.06 (Cys S-*C*H_2_CH_3_), 33.10 (Cys β-CH2), 52.70 (Cys α-CH), 169.99 and 172.96 (2 x CO).

#### N-Ac-Cys(Allyl)-OH (19)

Yield: 97%; R_*f*_ = 0.62, CH_2_Cl_2_:MeOH (9:1); ^1^H-NMR (300 MHz, 298.2 K, DMSO-d_6_): δ = 1.81 (s, 3H, Ac), 2.59–2.80 (m, 2H, Cys β-CH2), 3.13–3.15 (d, 2H, Cys S-C*H*_*2*_CHCH_2_), 4.35–4.39 (m, 1H, Cys α-CH), 5.07–5.12 (m, 2H, Cys S-CH_2_CHC*H*_*2*_), 5.63–5.80 (m, 1H, Cys S-CH_2_C*H*CH_2_), 8.20–8.23 (d, 1H, Cys NH), 12.60 (br s, 1H, COO*H*); ^13^C-NMR (300 MHz, 298.2 K, DMSO-d_6_): δ = 23.03 (Ac), 32.18 (Cys β-CH2), 34.66 (Cys S-*C*H_2_CHCH_2_), 52.35 (Cys α-CH), 118.09 (Cys S-CH_2_CH*C*H_2_), 134.88 (Cys S-CH_2_ *C*HCH_2_), 169.94 and 172.94 (2 x CO).

#### N-Ac-Met(Se)-OH (23)

Yield: 99%; R_*f*_ = 0.11, AcOEt:MeOH (9:1); ^1^H-NMR (300 MHz, 298.2 K, DMSO-d_6_): δ = 0.92 (s, 3H, Met Se-CH_3_), 1.33 (m, 2H, Met γ-CH2), 1.84 (s, 3H, Ac), 1.95–2.15 (m, 2H, Met β-CH2), 4.48–4.55 (m, 1H, Met α-CH), 8.05–8.08 (d, 1H, Met NH); ^13^C-NMR (300 MHz, 298.2 K, DMSO-d_6_): δ = 11.91 (Se-CH_3_), 22.74 (Met β-CH2), 23.35 (Ac), 25.42 (Met γ-CH2), 57.18 (Met α-CH), 174.57 and 179.11 (2 x CO).

#### General method for alkylation

A solution of commercially available NAC (**15**) (6.13 mmol) and KOH (13.48 mmol) in MeOH (1 mL) was added with propargylic bromide (7.36 mmol) and left for 1 h under reflux. After evaporation of the solvent, the residue was taken up with water, acidified with HCl 1N (pH = 2), then extracted with AcOEt. The organic layer was dried over anhydrous Na_2_SO_4_ and evaporated under vacuum to give product as oil, which were used without further purification.

#### N-Ac-Cys(Propargyl)-OH (20)

Yield: 99%. R_*f*_ = 0.1, CH_2_Cl_2_:MeOH (9:1); ^1^H-NMR (300 MHz, 298.2 K, DMSO-d_6_): δ = 1.85 (s, 3H, Ac), 2.78 (d, 2H, Cys β-CH_2_), 3.18 (s, 1H, S-CH_2_CC*H*), 3.33 (s, 2H, S-CH_2_CCH), 4.39 (m, 1H, Cys α-CH), 8.24–8.26 (d, 1H, NH). ^13^C-NMR (300 MHz, 298.2 K, DMSO-d_6_): δ = 22.52 (Cys S-*C*H_2_CCH), 23.18 (Ac), 32.04 (Cys β-CH2), 52.45 (Cys α-CH), 73.91 (Cys S-CH_2_C*C*H), 77.03 (Cys S-CH_2_ *C*CH), 178.66 and 178.81 (2 x CO).

#### General method for coupling reaction

Suitably protected compounds (**16**–**20**, **23**–**26**, and **28**) (5.1 mmol) were dissolved in DMF (2 mL) and DCM (10 mL), and then added with DCC (5.1 mmol). After 1 h under stirring at room temperature, the reaction mixture was added with DMAP (0.16 mmol) and carvacrol (5.1 mmol) and stirred overnight at room temperature. After reduced pressure filtration, the solvent was evaporated and the residue was extracted with AcOEt/NaCl ss. The organic layer was dried over anhydrous Na_2_SO_4_ and evaporated under vacuum. Chromatographic purification with DCM/AcOEt 1:1 as eluant provided the desired compounds in good yields (**2–7**, **9–10**, **21**, and **27**).

#### N-Ac-Cys(Methyl)-CAR (2)

Yield: 30%. R_*f*_ = 0.62, DCM:AcOEt (1:1); ^1^H-NMR (300 MHz, 298.2 K, CDCl_3_): δ = 1.19–1.23 (d, 6H, 2 x CH_3_, *i*-Pr), 2.04 (s, 3H, SCH_3_), 2.16 (s, 3H, CH_3_), 2.22 (s, 3H, Ac), 2.78–2.91 (m, 1H, *i*-Pr), 3.08–3.21 (m, 2H, Cys β-CH_2_), 5.08–5.15 (m, 1H, Cys α-CH), 6.55–6.57 (d, 1H, NH), 6.68–7.16 (m, 3H, Ar). ^13^C-NMR (300 MHz, 298.2 K, CDCl_3_): δ = 15.75 (SCH_3_), 16.69 (CH_3_), 23.29 (Ac), 24.13–24.29 (2x CH_3_, *i*-Pr), 33.91 (CH *i*-Pr), 36.58 (Cys β-CH_2_), 52.25 (Cys α-CH), 113.26–148.50 (Ar), 169.83 and 170.66 (2 x CO).

#### N-Ac-Cys(Ethyl)-CAR (3)

Yield: 32%; R_*f*_ = 0.67, DCM:AcOEt (1:1); ^1^H-NMR (300 MHz, 298.2 K, CDCl_3_): δ = 1.21 (d, 6H, 2 x CH_3_, *i*-Pr), 1,29 (t, 3H, SCH_2_C*H*_*3*_), 2.08 (s, 3H, CH_3_), 2.15 (s, 3H, Ac), 2.60–2.68 (m, 2H, SC*H*_*2*_CH_3_), 2.82–3.07 (m, 2H, Cys β-CH_2_), 5.08–5.12 (m, 1H, Cys α-CH), 6.38–6.41 (d, 1H, NH), 6.87–7.16 (m, 3H, Ar). ^13^C-NMR (300 MHz, 298.2 K, CDCl_3_): δ = 14.97 (SCH_2_ *C*H_3_), 16.13 (CH_3_), 23.40 (Ac), 24.13 (CAR, 2 x CH_3_, *i*-Pr), 27.15 (S*C*H_2_CH_3_), 33.78 (CH *i*-Pr), 34.05 (Cys β-CH_2_), 52.31 (Cys α-CH), 119.67–148.46 (Ar), 162.81 and 170.63 (2 x CO).

#### N-Ac-Cys(Allyl)-CAR (4)

Yield: 35%; R_*f*_ = 0.79, DCM:AcOEt (1:1); ^1^H-NMR (300 MHz, 298.2 K, CDCl_3_): δ = 1.19–1.21 (d, 6H, *i*-Pr), 2.09 (s, 3H, Ac), 2.18 (s, 3H, CH_3_), 2.78–2.83 (m, 1H, *i*-Pr), 2.85–3.08 (m, 2H, Cys β-CH_2_), 3.17 (d, 2H, SC*H*_*2*_CHCH_2_), 5.02–5.09 (m, 1H, Cys α-CH), 5.10–5.19 (m, 2H, SCH_2_CHC*H*_*2*_), 5.71–5.82 (m, 1H, SCH_2_C*H*CH_2_), 6.24–6.27 (d, 1H, NH), 6.83–7.18 (m, 3H, Ar). ^13^C-NMR (300 MHz, 298.2 K, CDCl_3_): δ = 15.76 (CH_3_), 23.30 (Ac), 24.14–24.29 (2 x CH_3_, *i*-Pr), 32.25 (CH *i*-Pr), 32.93 (Cys β-CH_2_), 35.59 (S-C*H*_*2*_CHCH_2_), 58.62 (Cys α-CH), 118.52 (SCH_2_CH*C*H_2_), 121.44–131.33 (Ar), 133.59 (S*C*H_2_CHCH_2_), 170.95 and 199.09 (2 x CO).

#### N-Ac-Met-CAR (5)

Yield: 47%; R_*f*_ = 0.74, DCM:AcOEt (1:1); ^1^H-NMR (300 MHz, 298.2 K, CDCl_3_): δ = 1.20–1.25 (d, 6H, 2 x CH_3_, *i*-Pr), 2.04 (s, 3H, Ac), 2.08 (s, 3H, CH_3_), 2.11 (s, 3H, SCH_3_), 2.11–2.44 (m, 2H, Met β-CH_2_), 2.63–2.68 (m, 2H, Met γ-CH_2_), 2.82–2.91 (m, 1H, CH *i*-Pr), 4.97–5.04 (m, 1H, Met α-CH), 6.25–6.27 (d, 1H, NH), 6.85–7.16 (m, 3H, Ar). ^13^C-NMR (300 MHz, 298.2 K, CDCl_3_): δ = 15.81 (SCH_3_), 16.10 (CH_3_), 23.39 (Ac), 24.13 (2 x CH_3_, *i*-Pr), 30.39 (CH *i*-Pr), 32.06 (Met γ-CH_2_), 33.79 (Met β-CH_2_), 51.99 (Met α-CH), 119.64–149.02 (Ar), 170.52 and 170.90 (2 x CO).

#### N-Ac-Met(Se)-CAR (6)

Yield: 35%; R_*f*_ = 0.65, DCM:AcOEt (1:1); ^1^H-NMR (300 MHz, 298.2 K, CDCl_3_): δ = 1.20–1.23 (d, 6H, 2 x CH_3_, *i*-Pr), 2.04 (s, 3H, Ac), 2.08 (s, 3H, CH_3_), 2.13 (s, 3H, SeCH_3_), 2.17–2.46 (m, 2H, Met β-CH_2_), 2.64–2.69 (m, 2H, Met γ-CH_2_), 2.85–2.90 (m, 1H, CH *i*-Pr), 4.97–5.04 (m, 1H, Met α-CH), 6.25–6.27 (d, 1H, NH), 6.85–7.16 (m, 3H, Ar). ^13^C-NMR (300 MHz, 298.2 K, CDCl_3_): δ = 4.64 (SeCH_3_), 16.12 (CH_3_), 20.59 (Met β-CH_2_), 23.45 (Ac), 24.12 (2 x CH_3_, *i*-Pr), 33.34 (Met γ-CH_2_), 33.79 (CH *i*-Pr), 52.79 (Met α-CH), 119.65–149.0 (Ar), 170.34 and 170.79 (2 x CO).

#### LA-CAR (7)

Yield: 38%; R_*f*_ = 0.81, DCM; ^1^H-NMR (300 MHz, 298.2 K, CDCl_3_): δ = 1.21–1.24 (d, 6H, 2 x CH_3_, *i*-Pr), 1.54–1.97 (m, 8H, 4 x CH_2_), 2.13 (s, 3H, CH_3_), 2.45–2.52 (m, 1H, CH *i*-Pr), 2.57–2.62 (t, 2H, CH_2_), 2.85–2.89 (m, 1H, SCH), 3.11–3.20 (m, 2H, SCH_2_), 6.84–7.15 (m, 3H, Ar). ^13^C-NMR (300 MHz, 298.2 K, CDCl_3_): δ = 16.12 (CAR CH_3_), 24.16 (2 x CH_3_, *i*-Pr), 25.03 (CH_2_), 29.07 (CH_2_), 33.79 (CH *i*-Pr), 34.27 (CH_2_), 34.87 (CH_2_), 38.76 (SCH_2_), 40.48 (CH_2_), 56.56 (SCH), 119.99–149.43 (Ar), 171.99 (CO).

#### N-Ac-Cys(Propargyl)-CAR (9)

Yield: 42%; R_*f*_ = 0.23, DCM:AcOEt (1:1); ^1^H-NMR (300 MHz, 298.2 K, CDCl_3_): δ = 1.21–1.23 (d, 6H, 2 x CH_3_, *i*-Pr), 1.63–1.88 (m, 2H, Cys β-CH_2_), 2.08 (s, 3H, Ac), 2.15 (s, 3H, CH_3_), 2.31–2.33 (s, 1H, SCH_2_CC*H*), 2.84–2.89 (m, 1H, *i*-Pr), 3.13–3.38 (2H, m, SC*H*_*2*_CCH), 5.14 (m, 1H, Cys α-CH), 6.49–6.51 (d, 1H, NH), 6.89–7.15 (m, 3H, Ar). ^13^C-NMR (300 MHz, 298.2 K, CDCl_3_): δ = 16.15 (CH_3_), 20.31 (S*C*H_2_CCH), 23.36 (Ac), 24.13 (2 x CH_3_, *i*-Pr), 33.78 (Cys β-CH_2_), 34.14 (CH *i*-Pr), 52.11 (Cys α-CH), 72.50 (SCH_2_C*C*H), 79.45 (SCH_2_ *C*CH), 119.68–149.04 (Ar), 169.58 and 170.41 (2 x CO).

#### OTC-CAR (10)

Yield: 33%; R_*f*_ = 0.78, DCM:AcOEt (9:1); ^1^H-NMR (300 MHz, 298.2 K, CDCl_3_): δ = 1.21–1.23 (d, 6H, 2 x CH_3_, *i*-Pr), 2.11 (s, 3H, CH_3_), 2.85–2.89 (m, 1H, *i*-Pr), 3.77–3.87 (m, 2H, OTC β-CH_2_), 4.66–4.71 (m, 1H, OTC α-CH), 6.86–7.17 (m, 3H, Ar), 6.91 (s, 1H, NH). ^13^C-NMR (300 MHz, 298.2 K, CDCl_3_): δ = 16.00 (CH_3_), 24.12 (2 x CH_3_, *i*-Pr), 32.28 (CH, *i*-Pr), 33.80 (OTC β-CH_2_), 56.44 (OTC α-CH), 119.47–148.82 (Ar), 168.83 and 175.08 (2 x CO).

#### N-Ac-Cys(Trt)-CAR (21)

Yield: 33%; R_*f*_ = 0.18, DCM:AcOEt (1:1); ^1^H-NMR (300 MHz, 298.2 K, CDCl_3_): δ = 1.20 (d, 6H, *i*-Pr), 1.99 (s, 3H, Ac), 2.08 (s, 3H, CH_3_), 2.67–2.95 (m, 2H, Cys β-CH_2_), 4.81–4.88 (m, 1H, Cys α-CH), 6.57 (d, 1H, NH), 6.88–7.46 (m, 18H, Ar). ^13^C-NMR (300 MHz, 298.2 K, CDCl_3_): δ = 16.15 (CH_3_), 23.34 (Ac), 24.13 (2x CH_3_, *i*-Pr), 34.16 Cys (β-CH_2_), 51.48 (Cys α-CH), 67.34 (C Trt), 119.76–149.11 (Ar), 168.79 and 170.34 (2 x CO).

#### Boc-TCA-CAR (27)

Yield: 40%; R_*f*_ = 0.64, DCM; ^1^H-NMR (300 MHz, 298.2 K, CDCl_3_): δ = 1.21–1.25 (d, 6H, 2 x CH_3_, *i*-Pr), 1.48 (s, 9H, Boc), 2.16 (s, 3H, CH_3_), 2.85–2.90 (m, 1H, *i*-Pr), 3.43–3.49 (m, 2H, TCA β-CH_2_), 4.50–4.75 (m, 2H, TCA δ-CH_2_), 4.96–5.16 (m, 1H, TCA α-CH), 6.85–7.15 (m, 3H, Ar). ^13^C-NMR (300 MHz, 298.2 K, CDCl_3_): δ = 16.07 (CH_3_), 24.16 (2 x CH_3_, *i*-Pr), 28.55 (Boc), 33.67 (CH *i*-Pr), 35.01 (TCA β-CH_2_), 48.39 (TCA δ-CH_2_), 62.02 (TCA α-CH), 81.53 (Boc), 119.44–148.39 (Ar), 153.56 (OCONH), 169.23 (CO).

### Removal of protecting groups

#### NAC-CAR (1)

A solution of **21** (100 mg, 0.17 mmol) in DCM (0.3 mL) was added with TIPS (0.27 mL, 0.21 mmoL) and TFA (0.52 mL, 6.8 mmol) under nitrogen atmosphere. The reaction mixture was stirred for 48 h at room temperature and then dried under vacuum. The crude was purified by chromatographic column using DCM/MeOH 9:1 as eluant to afford the final deprotected compound **1**. Yield: 88%; ^1^H-NMR (300 MHz, 298.2 K, CDCl_3_): δ = 1.21–1.23 (d, 6H, *i*-Pr), 2.07 (s, 3H, Ac), 2.15 (s, 3H, CH_3_), 2.87 (m, 1H, *i*-Pr), 3.17–3.23 (m, 2H, Cys β-CH_2_), 5.16–5.19 (m, 1H, Cys α-CH), 6.57–6.60 (d, 1H, NH), 6.87–7.17 (m, 3H, Ar). ^13^C-NMR (300 MHz, 298.2 K, CDCl_3_): δ = 16.19 (CH_3_), 23.35 (Ac), 24.12 (2 x CH_3_, *i*-Pr), 27.21 (CH *i*-Pr), 33.79 (Cys β-CH_2_), 53.91 (Cys α-CH), 119.70–148.97 (Ar), 169.08 and 170.32 (2 x CO).

#### TFA·TCA-CAR (8)

A solution of **27** (327 mg, 0.9 mmol) in TFA (1.35 mL) was left for 2 h under stirring at room temperature [32]. Yield: 100%; R_*f*_ = 0.21, DCM; ^1^H-NMR (300 MHz, 298.2 K, CDCl_3_): δ = 1.21–1.25 (d, 6H, 2 x CH_3_, *i*-Pr), 2.09 (s, 3H, CH_3_), 2.84–2.90 (m, 1H, *i*-Pr), 3.54–3.64 (m, 2H, TCA β-CH_2_), 4.35–4.52 (m, 2H, TCA δ-CH_2_), 5.04–5.08 (m, 1H, TCA α-CH), 6.85–7.16 (m, 3H, Ar), 9.48 (s, 2H, NH_2_). ^13^C-NMR (300 MHz, 298.2 K, CDCl_3_): δ = 15.69 (CH_3_), 24.00 (2 x CH_3_, *i*-Pr), 33.45 (CH *i*-Pr), 33.77 (TCA β-CH_2_), 49.09 (TCA δ-CH_2_), 62.82 (TCA α-CH), 119.44–148.99 (Ar), 166.24 (CO).

### Antimicrobial activity

Two Gram-positive (*S*. *aureus* ATCC 29213 and *S*. *epidermidis* ATCC 35984) and two Gram-negative (*E*. *coli* ATCC 8739 and *P*. *aeruginosa* ATCC 9027) strains, and one fungal strain (*C*. *albicans* ATCC 10231) were used for the detection of antibacterial activity of carvacrol, NAC, Ac-Cys(Allyl)-OH, and carvacrol codrugs **1**–**10**. All tested compounds were dissolved in 20% DMSO to prepare the stock solution (100 mg/mL). The final concentration of DMSO in each well appeared to be not toxic for all studied microorganisms, as confirmed by the solvent control test included in the study.

The effects of carvacrol codrugs **1**–**10** and reference compounds on planktonic cells were evaluated by Minimum Inhibitory Concentration (MIC) and Minimum Bactericidal Concentration (MBC) determination using the broth microdilution method according to EUCAST guidelines [33]. Bacterial suspensions, grown in Mueller–Hinton Broth (MHB) at logarithmic phase, were incubated on microtiter plates at a concentration of 5 x 10^5^ CFU/mL, with several dilutions (0.07–40 mg/mL) for 24 h at 37°C. The MIC was defined as the lowest concentration of substances giving a complete inhibition of visible growth in comparison with a control well, and the MBC was determined as the lowest concentration at which no bacterial growth occurred on Mueller–Hinton Agar plates.

The MIC detection of *C*. *albicans* ATCC 10231 was performed using the broth microdilution method according to EUCAST guidelines [34] in RPMI 1640 plus 2% glucose with a final inoculum of 1–5 x 10^5^ CFU/mL for 24–48 h at 37°C. The Minimum Fungicidal Concentration (MFC) was determined as the lowest concentration of substances at which no fungal growth occurred on Sabouraud agar plates.

Data were obtained from at least three independent experiments performed in duplicate. The susceptibility of the bacterial strains against ciprofloxacin was used as an internal standard during MIC determinations.

#### Antibiofilm assay

The efficacy on established biofilm was evaluated by determining Biofilm Inhibitory Concentration (BIC) and Biofilm Eradication Concentration (BEC) according to the method described by Johnson *et al*. [35] with some modifications. Bacterial suspensions, grown in Tryptic soy broth supplemented with 0.5% (v/v) glucose at logarithmic phase, as well as *C*. *albicans* grown in RPMI 1640 plus 2% glucose, were incubated on flat-bottomed microtiter plates at a concentration of 5 x 10^5^ CFU/mL. After 24 h of incubation at 37°C, the planktonic cells were gently removed and wells were washed with sterile phosphate-buffered saline solution (PBS) pH 7.3 and filled with carvacrol, carvacrol codrug **4**, Ac-Cys(Allyl)-OH with dilutions ranging from the MIC values to a maximum concentration of 50 mg/mL. The OD_600_ was measured at time 0 and after incubation for 24 h at 37°C. The BIC values were determined as the lowest concentrations where no growth occurred in the supernatant fluid, confirmed by no increase in optical density compared with the initial reading. The BEC values were determined as the lowest concentrations at which no bacterial growth occurred on Tryptic soy agar for bacteria and on Sabouraud agar for *C*. *albicans*. Data were obtained from at least three independent experiments performed in duplicate.

#### Hemolytic Activity

Experimental protocols to study hemolysis on human red cells were approved by Ethics Committee of the University “G. d’Annunzio” Chieti-Pescara. Human red cells were from two volunteers, who signed the informed consent for this study.

Hemolytic activity of carvacrol codrugs **1**–**10** was tested against human red blood cells (h-RBC). Fresh human blood, collected with EDTA, was centrifuged at 3000 rpm for 5 min then washed three times with PBS pH 7.3. Red blood cells were diluted to 4% in PBS and incubated with different codrugs concentrations ranging from 25 to 0.05 mg/mL. After 1 h of incubation at 37°C, the suspensions were sedimented by centrifugation and the release of hemoglobin was determined by absorbance measurement at 405 nm and compared with a 0% hemolysis control (PBS) and a 100% hemolysis control (PBS with 1% v/v Triton X-100). The percentage of hemolysis was calculated using the following equation:

$$Hemolysis\;(\%)\;=\;[\left(OD_{405}sample\;-\;OD_{405}0\%\;lysis\;control\right)/\left(OD_{405}100\%\;lysis\;control\;-\;OD_{405}0\%\;lysis\;contro\right)]\;X\;100$$

#### Transmission electron microscopy (TEM)

For transmission electron microscopy, microrganisms were fixed with 2.5% glutaraldehyde in 0.1 M cacodylate buffer, postfixed with 1% OsO_4_, dehydrated in graded ethanol and embedded in Epon 812. Ultrathin sections (60 to 80 nm) were then mounted on 200-mesh copper grids and stained with uranyl acetate and lead citrate for ultrastructural observation. Sections were photographed using a Philips 268 D electron microscope (FEI).

### Pharmacokinetic studies

#### HPLC–UV assays

The HPLC system consisted of a Waters 600 HPLC pump (Waters Corporation, Milford, MA, USA), equipped with a Waters 2996 photodiode array detector. The column was a Kinetex RP-C8 column (3.0 x 150 mm, 5 μm). The mobile phase consisted of 0.1% TFA in acetonitrile/0.1% TFA in 95% H_2_O and 5% acetonitrile 50:50, under isocratic conditions at a flow rate of 1 mL/min. The UV detector was set at a length of 220 nm.

#### Solubility

The water solubility was determined by stirring an excess of the compound in 1 mL of water for 15 min at room temperature. Then the mixture was filtered using a Millipore filter (0.45 μm) and the concentration of the compound in its saturated solution was determined by HPLC analysis [36].

#### Lipophilicity

The calculated clogP values were determined using ACD LogP software package, version 4.55 (Advanced Chemistry Development Inc., Toronto, Canada).

#### Kinetics of chemical hydrolysis

The chemical hydrolysis rate of compound **4** was studied in solution of 0.02 M hydrochloric acid buffer of pH 1.3, as non-enzymatic simulated gastric fluid (SGF), and a 0.02 M phosphate buffer of pH 7.4 at 37°C, as simulated intestinal fluid (SIF). Reactions were initiated by adding 1 mL of 10^-4^ M stock solution (in acetonitrile) of the compound to 10 mL of the appropriate aqueous buffer solution, containing 20% acetonitrile. At appropriate time intervals, samples of 20 μL were withdrawn and analyzed by HPLC. Pseudo-first-order rate constants (k_obs_) for the hydrolysis of the compounds were then calculated from the slopes of the linear plots of log (% residual compound) against time. The experiments were run in triplicate and the mean values of the rate constants were calculated [37].

#### Kinetics of enzymatic hydrolysis

The enzymatic hydrolysis was evaluated in human and rat plasma. Stock solution of drug was prepared in methanol (1 mg/mL) which did not have effects on the enzymatic degradation (data not shown). A volume of 200 μL of this solution was added with pre-heated (37°C) plasma fractions (4 mL) previously diluted with 0.02 M phosphate buffer (pH 7.4) to give a final volume of 5 mL (80% plasma). Aliquots (100 μL) were taken at various times and deproteinized by mixing with 200 μL of 0.01 M HCl in methanol. After centrifugation for 5 min at 5000 × g, 10 μL of the supernatant layer were analysed by chromatography as described above. The amounts of remaining intact compound were plotted as a function of incubation time [38].

#### Drug stability testing in intestinal fluids

To assess enzymatic stability, hydrochloric buffer with pepsin (10 or 40 mg/mL) and phosphate buffer with pancreatin (10 or 40 mg/mL) were used. Buffer solution (250 μL) was preincubated at 37°C and 50 μL of drug stock solution (50 μM in ethanol and FASSIF 1:9) were added and shaken at 37°C and 650 rpm. Samples of 100 μL were withdrawn at various times and 100 µL of ice-cold acetonitrile containing 0.5 v/v% formic acid were added to stop enzymatic activity [39]. Samples were vortexed and centrifuged at 2°C and 10000 rpm for 10 min. The drug content in the supernatant was analyzed by HPLC.

#### PAMPA Method

Parallel Artificial Membrane Permeability Assay (PAMPA) was used to measure the permeability coefficient (*P*_*e*_) of codrug **4** through the artificial membrane to predict oral absorption. The carvacrol codrug **4** permeability was determined using PAMPA-GI protocols already developed [37]. Briefly, donor and acceptor plates were assembled to form a “sandwich” and incubated at room temperature for 18 h.

*P*_*e*_ can be calculated from the equation below:

$$Pe=\left\{\frac{V_{D}\cdot V_{A}}{\left(V_{D}+V_{A}\right)\cdot A\cdot t}\cdot \ln\left(1-\frac{{\left[drug\right]}_{acceptor}}{{\left[drug\right]}_{equilibrium}}\right)\right\}$$ (1)

where *P*_*e*_ is the effective permeability coefficient (cm × s^-1^), *V*_*D*_ is the volume of the donor compartment (0.15 cm^3^) and *V*_*A*_ is the volume of the acceptor compartment (0.30 cm^3^), A is the effective filter area (0.28 cm^2^), *t* is the incubation time for the assay (s), *[drug]*_*acceptor*_ is the concentration of the compound in the acceptor compartment at assay completion, and *[drug]*_*equilibrium*_ is the concentration of the compound at theoretical equilibrium.

## Results and Discussion

### Synthesis of carvacrol codrugs 1–10

**Fig 2:**
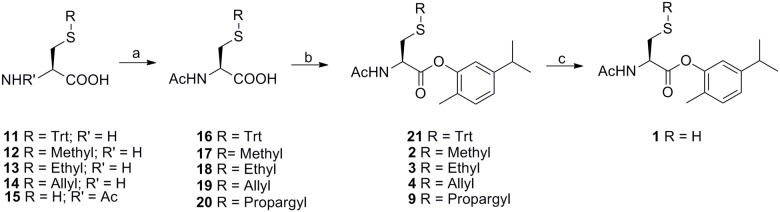
Synthesis of carvacrol codrugs 1–4 and 9. Reagents and conditions: a) Ac_2_O, AcOH, 4 h, rt for compounds **11–14**; KOH, Propargyl bromide, in dry MeOH, 1 h, 60°C (reflux) for compound **15**; b) DCC in DMF/DCM, 1 h, rt, then carvacrol, DMAP, 15 h, rt; c) TIPS, TFA in DCM, 48 h, rt under nitrogen atmosphere.

**Fig 3:**
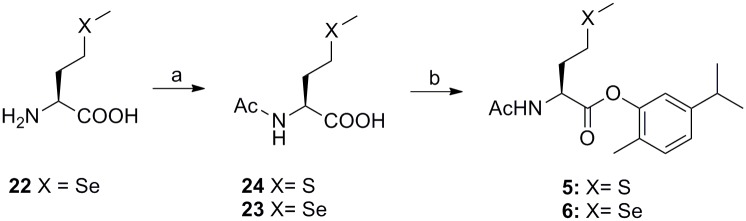
Synthesis of carvacrol codrugs 5–6. Reagents and conditions: a) Ac_2_O, AcOH, 4 h, rt for compound **22**; b) DCC in DMF/DCM, 1 h, rt, then carvacrol, DMAP, 15 h, rt.

**Fig 4:**
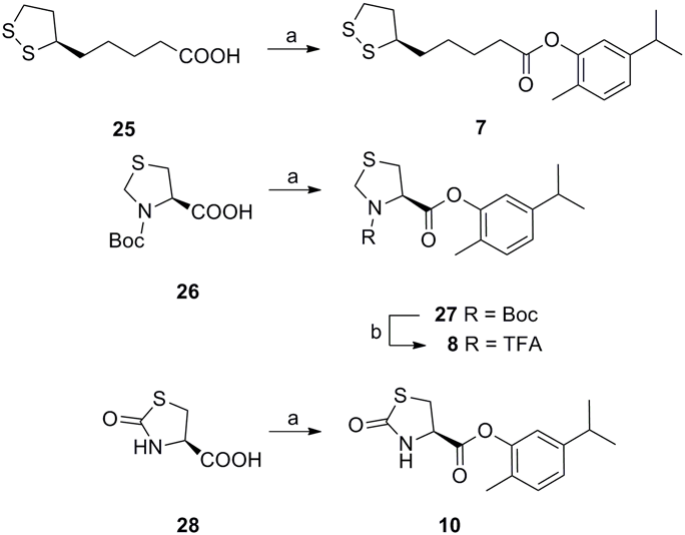
Synthesis of carvacrol codrugs 7–8 and 10. Reagents and conditions: a) DCC in DMF/DCM, 1 h, rt, then carvacrol, DMAP, 15 h, rt for compounds **25–28**; b) TFA, 2 h, rt for compound **27**.

Carvacrol codrugs **1–10** were synthesized as outlined in Figs 2–4 employing solution phase procedures by elongation of the suitably protected Cys aminoacid chain in the C direction. Acetylation of aminic groups of cysteine derivatives **11–14**, and **22** was performed as previously described [40] to obtain compounds **16–19**, and 23 in good yields (Figs 2–3). Alkylation of commercial NAC (**15**) was performed using propargyl bromide in dry MeOH for 1 h at 60°C in basic conditions to afford the derivative 20 in good yield (Fig 1).

The syntheses of the carvacrol codrugs **1–10** were successfully carried out by using N,N’-dicyclohexylcarbodiimide (DCC), which proves to be an effective catalyst for the conversion of carboxylic acids to esters and amides (Figs 2–4). Particularly, compounds **16–20**, **23–26** and **28** were treated with DCC in DMF/DCM for 1 h at room temperature; after the addition of carvacrol and DMAP, the mixture was left under stirring for 15 h at room temperature. The carvacrol codrugs **2–7** and **9–10** were obtained and purified on silica gel using DCM/AcOEt as eluant (yields 30–47%).

A further step was necessary to obtain the final compounds **1** and **8**; in case of carvacrol codrug **1** the deprotection of the cysteine-SH group was reached when the corresponding protected precursor **21** was treated with TIPS, TFA in DCM for 48 h under nitrogen atmosphere at room temperature (yield 88%) (Fig 2); the removal of the Boc protecting group on **27** by treatment with TFA for 2 h at room temperature afforded the corresponding trifluoracetate 8 in quantitative yield (Fig 4). Before performing biological studies, all carvacrol codrugs **1–10** were fully characterized by ^1^H-NMR, ^13^C-NMR spectra and the purity was checked by HPLC analysis. A grade of purity higher than 98% after purification was obtained for all the final compounds.

### Antimicrobial activity of carvacrol codrugs 1–10

In this study, the antimicrobial activity of carvacrol codrugs **1–10**, carvacrol, NAC, and Ac-Cys(Allyl)-OH (the last three as reference compounds) was evaluated against pathogenic microorganisms and the results are outlined in Table 1.

**Table 1:** MIC^a^ and MBC^a^ values of carvacrol codrugs 1–10 against bacterial strains and *C*. *albicans*.

Compounds (mg/mL)	*S. aureus* ATCC 29213		*S. epidermidis* ATCC 35984		*E. coli* ATCC 8739		*P. aeruginosa* ATCC 9027		*C. albicans* ATCC 10231	
	MIC	MBC	MIC	MBC	MIC	MBC	MIC	MBC	MIC	MFC^a^
Carvacrol	0.6	1.25	0.3	1.25	0.6	1.25	5	10	0.3	0.6
NAC	5	20	5	5	5	5	5	5	20	80
Ac-Cys(Allyl)-OH	5	5	5	5	2.5	2.5	10	10	2.5	2.5
**1**	10	10	5	20	10	10	10	10	0.3	0.3
**2**	10	10	2.5	10	10	10	10	10	5	5
**3**	10	10	10	10	10	10	10	10	2.5	2.5
**4**	2.5	2.5	2.5	2.5	2.5	2.5	5	10	1.25	1.25
**5**	10	10	10	10	10	10	5	10	5	5
**6**	10	10	5	10	5	10	5	10	0.15	0.6
**7**	10	10	10	10	5	10	5	10	2.5	5
**8**	5	10	2.5	5	5	5	10	10	0.6	0.6
**9**	10	10	5	10	10	10	10	10	1.25	2.5
**10**	10	10	10	10	10	10	10	10	2.5	2.5

^a^ Abbreviations: MIC, Minimum Inhibitory Concentration; MBC, Minimum Bactericidal Concentration; MFC, Minimum Fungicidal Concentration.

When considering antimicrobial potency against Gram positive and Gram negative, carvacrol codrug **4** resulted the most active with bacteriostatic and bactericidal values equal to 2.5 mg/mL for all bacterial strains except for *P*. *aeruginosa* ATCC 9027, for which MIC and MBC values were 5 mg/mL and 10 mg/mL, respectively. We observed a lower antibacterial potency for all the other carvacrol codrugs (MIC values ranging from 2.5 mg/mL to 10 mg/mL) (Table 1). We also tested the reference compounds: a) the antimicrobial effect exerted by NAC—MIC and MBC values ranged from 5 to 20 mg/mL for Gram positive and Gram negative tested bacteria—was similar to those reported by Aslam and Darouiche [41], suggesting that this amino acid may competitively inhibit bacterial utilization of cysteine or react via its sulfhydryl group with bacterial membranes; b) Ac-Cys(Allyl)-OH showed a better antimicrobial activity (MIC values from 2.5 to 10 mg/mL) respect to NAC probably due to the presence of the allyl moiety that might be a more offensive tool against bacteria, as previously reported [30]. In fact, the better antibacterial activity of Ac-Cys(Allyl)-OH compared to NAC is reflected in the corresponding carvacrol codrugs **4** and **1**, respectively, suggesting that the presence of the allyl-cysteine moiety in **4** improves the antibacterial properties. However, compound **4** showed a weaker potency against *P*. *aeruginosa* confirming the much lower outer membrane permeability than *E*. *coli* [42] probably due to the presence of efflux pumps that can transport anti-infective agents out of the periplasmatic space and to the reduced number and alteration of porins.

In this study, the same set of ten carvacrol codrugs was also tested against *C*. *albicans* ATCC 10231. Most of them, including carvacrol, showed antifungal activity with MIC values ranging from 0.15 mg/mL to 5 mg/mL (Table 1). In particular, carvacrol codrugs **6**, **1**, **8** displayed fungicidal activity with MIC values equal to 0.15, 0.3, and 0.6 mg/mL, respectively. On the other hand, NAC was inactive against this fungal strain (MIC 20 mg/mL) while Ac-Cy(Allyl)-OH displayed antifungal activity with MIC and MFC equal to 2.5 mg/mL. However, the carvacrol codrug **1**, containing the NAC moiety, showed a good antifungal activity while carvacrol codrug **4**, containing the allyl-cysteine, was less active. This antifungal behavior is opposite to the antibacterial one for these carvacrol codrug **1** and **4**. The antifungal activity is related to the presence and hydrophobicity of the carvacrol structure that is able to distribute into lipids of fungal membrane and interfere with its integrity causing fungal death. However, the overall lipophilicity of carvacrol codrugs **1** and **4** does not correlate with the results since codrug **1** is less hydrophobic (calculated LogP 2.66) than codrug **4** (calculated LogP 3.77) suggesting a potential involvement of the free thiol group in the antifungal activity of the carvacrol codrug. This consideration is in accordance with Bertling *et al*. [31] that support the hypothesis the thiol group is able to inactivate the gliotoxin produced by *C*. *albicans* by oxidation reactions. Also in the case of the best antifungal carvacrol codrug **6** (MIC 0.15 mg/mL) the hydrophobicity (calculated LogP 2.61) does not seem to be linked to the activity indicating that the presence of selenium might promote further interactions with the fungal membrane destabilizing it.

#### Antibiofilm effect of carvacrol codrug 4

BIC and BEC for carvacrol, Ac-Cys(Allyl)-OH, and the most active carvacrol codrug **4** were determined for all studied microorganisms (Table 2). BIC and BEC values of the three compounds for *S*. *aureus* ATCC 29213 were two-fold (2MIC) or four-fold (4MIC) greater than the concentration required to inhibit growth in suspension (MIC value); for *S*. *epidermidis* ATCC 35984 BIC and BEC values for carvacrol codrug **4** were 2MIC and 8MIC, equal to MIC and 2MIC for carvacrol, and 4MIC for Ac-Cys(Allyl)-OH; for *E*. *coli* ATCC 8739, Ac-Cys(allyl)-OH showed BIC and BEC values of 2MIC and 8MIC, while codrug **4** and carvacrol showed higher values: 8MIC, 16MIC, and 64MIC, respectively. BIC and BEC values of codrug **4**, carvacrol, and Ac-Cys(Allyl)-OH coincided with MIC and 2MIC values for *P*. *aeruginosa* ATCC 9027. As shown in Table 2, for *C*. *albicans* ATCC 10231 BIC and BEC values of codrug **4** were 8MIC, for carvacrol these coincided with MIC and MFC, and for Ac-Cys(Allyl)-OH BIC and BEC were 2MIC.

**Table 2:** BIC^a^ and BEC^a^ values of carvacrol codrug 4 against bacterial strains and *C*. *albicans*.

Compounds (mg/mL)	*S*. *aureus* ATCC 29213		*S*. *epidermidis* ATCC 35984		*E*. *coli* ATCC 8739		*P*. *aeruginosa* ATCC 9027		*C*. *albicans* ATCC 10231	
	BIC	BEC	BIC	BEC	BIC	BEC	BIC	BEC	BIC	BEC
Carvacrol	1.2	2.5	0.3	0.6	40	40	5	10	0.3	0.6
Ac-Cys(Allyl)-OH	10	20	20	20	5	20	10	20	5	5
**4**	5	10	5	20	20	40	5	10	10	10

^a^Abbreviations: BIC, Biofilm Inhibitory Concentration; BEC, Biofilm Eradicating Concentration.

Our results outlined that carvacrol possesses antimicrobial and antibiofilm properties but, except for *S*. *epidermidis* ATCC 35984 and *C*. *albicans* ATCC 10231, did not show an interesting and considerable effect on bacterial sessile phase respect to the planktonic one. *Escherichia coli* ATCC 8739 mature biofilm was more affected by Ac-Cys(Allyl)-OH and codrug **4** than carvacrol, suggesting that carvacrol is able to reduce bacterial biofilm formation interfering with the quorum sensing signaling mechanism but not with the mature biofilm, as reported by Burt *et al*. [43]. Probably, carvacrol alone is unable to penetrate the microbial biofilm matrix of *E*. *coli* while the conjugation to Ac-Cys(Allyl)-OH renders the codrug **4** able to promote the permeabilization and destabilization of the bacterial membrane.

#### Hemolytic Activity

**Fig 5:**
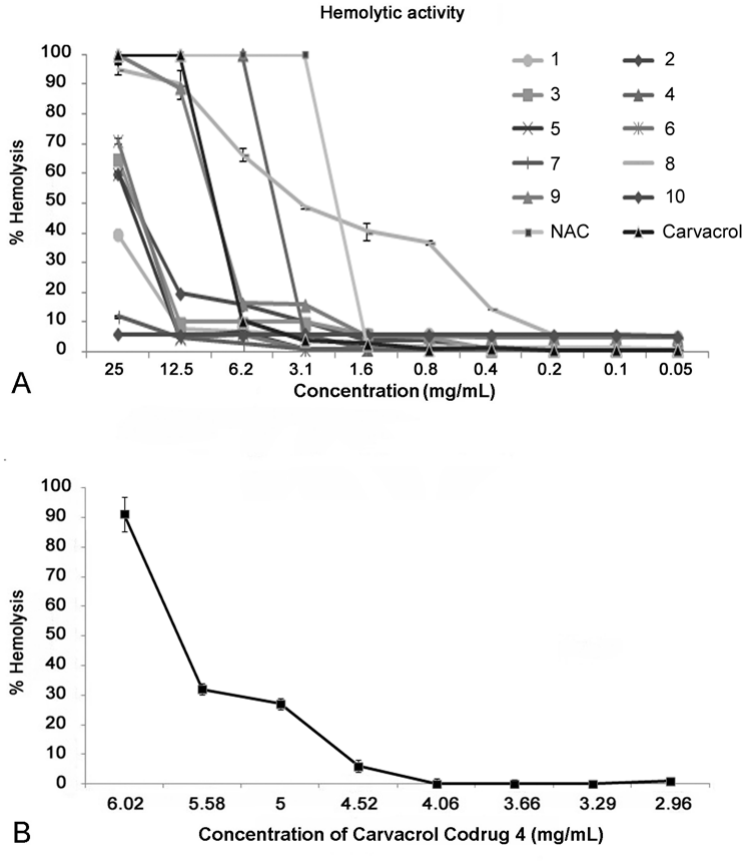
Hemolytic activity of carvacrol codrugs 1–10, CAR, and NAC (A) and in a wider range of values for carvacrol codrug 4 (B).

*In vitro* hemolytic assay represents a suitable screening tool to verify the *in vivo* toxicity to host cells. In the present study, the hemolytic activities of codrugs **1**–**10**, carvacrol and NAC, as reference compounds, were evaluated (Fig 5). All analyzed codrugs produced human blood hemolysis below 50% at their MIC values, except for codrugs **8** and **9** which showed hemolytic activity values over 50%. In particular, carvacrol codrugs **7** and **10** hemolytic activities were never more than 12% at the tested concentrations; codrug **1** never expressed hemolytic activity higher than 40% and for carvacrol codrugs **2**, **3**, **5**, and **6** the hemolytic activities were more than 50% only over 20 mg/mL of concentration. The carvacrol codrugs **4**, **8**, and **9** displayed hemolytic activity over 50% at 5 mg/mL, 3 mg/mL, and 10 mg/mL, respectively. These data correlate with the hydrophobicity parameters: the carvacrol codrugs **8** and **9**, showing the highest LogP values, resulted the most toxic compounds. Furthermore, carvacrol codrug **8** was obtained as trifluoracetate salt, which is substantially more toxic than hydrochloride salt even though they are both able to cross the intestinal membranes [44].

In particular, the hemolytic activity of carvacrol codrug **4** was evaluated in a wider range of values between 6.02–2.96 mg/mL to verify if the hemolysis occurs around the MIC value (2.5 mg/mL) for *S*. *aureus* ATCC 29213, *S*. *epidermidis* ATCC 35984, and *E*. *coli* ATCC 8739. Results showed that codrug **4** resulted not toxic at the MIC values for *S*. *aureus* ATCC 29213, *S*. *epidermidis* ATCC 35984, *E*. *coli* ATCC 8739, and *C*. *albicans* ATCC 1023.

#### Transmission Electron Microscopy of carvacrol codrug 4

**Fig 6:**
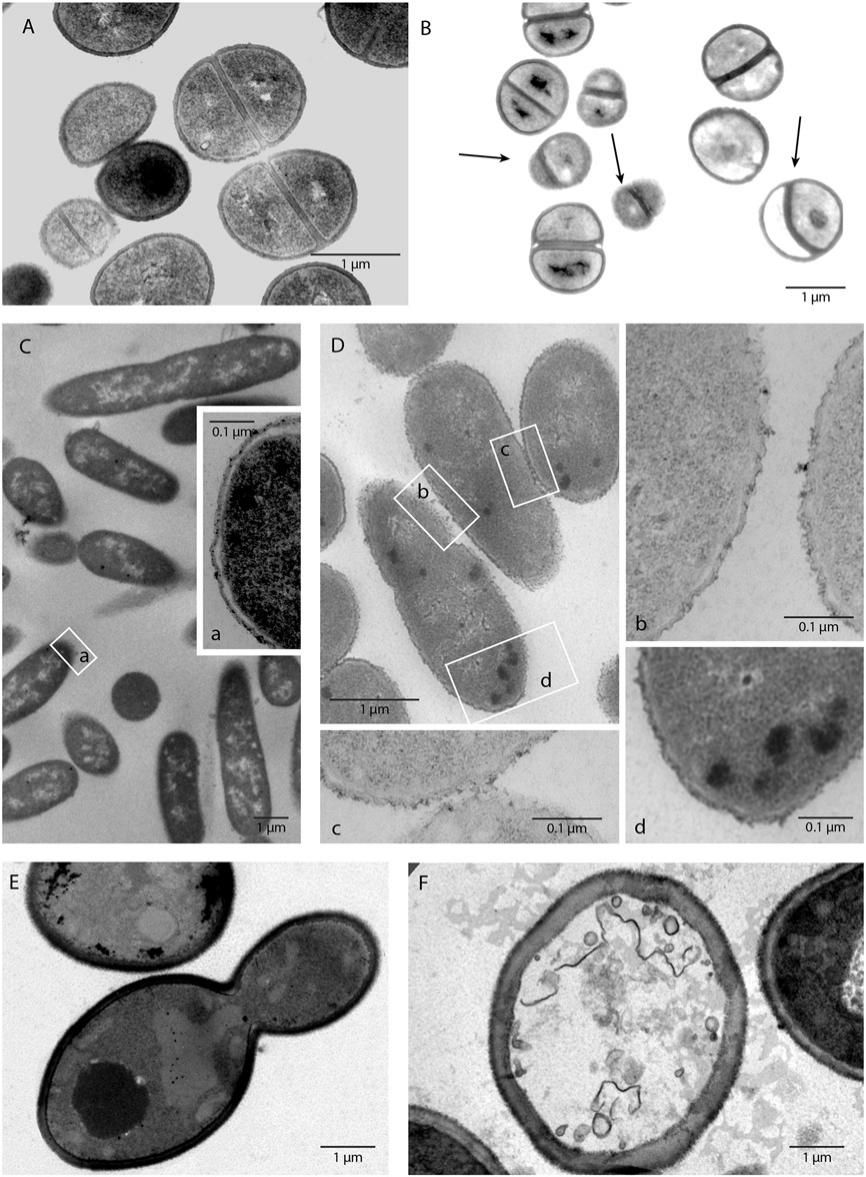
Transmission electron microscopy demonstrating the effects of carvacrol codrug 4 on *S*. *aureus* ATCC 29213 (B), *E*. *coli* ATCC 8739 (D), *C*. *albicans* ATCC 10231 (F), and untreated cultures (control), respectively (A, C, and E). Microorganisms incubated for 3 h in media containing 12.5 mg/mL [45] of carvacrol codrug **4** (B, D, and F). Irregular features of septa in *S*. *aureus* ATCC 29213 (B: arrows); numerous electron-dense bubbles protrude from the cell surface (higher magnification b 140000x and c 110000x) in *E*. *coli* ATCC 8739 treated (D) and electron-dense granules of the substance internalized into the cytoplasm (d 110000x); integrity of the membrane (a 110000x) in the controls (C); disintegration of membrane in *C*. *albicans* ATCC 10231 (F).

To elucidate the physiological effects of carvacrol codrug **4** against *S*. *aureus*, *E*. *coli*, and *C*. *albicans* transmission electron microscopy was utilized. As illustrated in Fig 6, the micrographs clearly demonstrated that the growth of *S*. *aureus*, *E*. *coli*, and *C*. *albicans* in media containing carvacrol codrug **4** at 12.5 mg/mL for 3 h generated profound changes in cell morphology [45]. We found that carvacrol codrug **4** had no effect on the envelope in *S*. *aureus*, but induced abnormal septum formation with irregular features (Fig 6B, arrows) probably with the same mechanism of action of some antibiotics such as penicillin [46], erythromycin [47], and vancomycin [48]. On the contrary, *E*. *coli* treated cells were evidently affected on the cell wall: alteration of outer membrane (Fig 3D), formation of membrane blebbing with numerous electron-dense bubbles protruding from the cell surface (Fig 6D–6c and 6b) and electron-dense granules internalized into the cytoplasm were observed (Fig 6D-d). In *C*. *albicans* we observed the disintegration of the membranes (Fig 6F) confirming that the carvacrol codrug **4** hydrophobicity allows it to interact with the fungal cell membrane and to interfere with its integrity. In fact, compound **4** could distribute into lipids of fungal membranes, rendering them more permeable and damaging their integrity, consequently causing mycelia death [49].

### Pharmacokinetic studies of carvacrol codrug 4

The pharmacokinetic parameters were studied only for carvacrol codrug **4** since it resulted the compound with the best antimicrobial activity.

Solubility is one of the most important parameters to reach the desired concentration of drug in systemic circulation for achieving required pharmacological response. Table 3 shows the water solubility of codrug **4** and its lipophilicity (LogP and cLogP). Our data revealed that codrug **4** has a low water solubility (0.15 mg/mL) and a cLogP value (3.77) similar to carvacrol (3.35), suggesting that this hydrophobic character could allow codrug **4** to interact with the bacterial membrane just like carvacrol.

**Table 3:** Physicochemical properties of carvacrol codrug 4.

Lipophilicity		Water Solubility^a^ (mg/mL)
LogP^a^	cLogP	
1.21 (± 0.04)	3.35	0.15 (± 0.01)

^a^Values are means of three experiments, standard deviation is given in parentheses.

The precondition of codrug strategy is that the codrug can be converted to the parent drugs to exert the pharmacological effect. For this purpose, the chemical and enzymatic stabilities of carvacrol codrug **4** were evaluated in three buffers (pH 1.3, 5.0, and 7.4), in simulated fluids (SGF and SIF) with different concentrations (10 mg/mL and 40 mg/mL) of enzymes (pepsin and pancreatin), and in human and rat plasma. As reported in Table 4, codrug **4** resulted more stable at pH 1.3 (t_½_ = 75.38 h) and pH 5.0 (t_½_ = 62.08 h), respect to pH 7.4 (t_½_ = 22.9 h). These values suggest that codrug **4** is able to pass unhydrolyzed the g.i. tract. In the presence of increasing concentrations of enzymes, the rate of hydrolysis of codrug **4** was significantly faster at pH 7.4 than 1.3; in fact, in the presence of pepsin (10 mg/mL) at pH 1.3, codrug **4** resulted stable with a t_½_ higher than 74 h (Table 4); however, with the increase in pepsin concentration the half-life of codrug **4** lowered (t_½_ = 5.79 h). On the other hand, in physiological buffer codrug **4** degraded with a half-life of 5.5 h and about 1 h in the presence of 10 and 40 mg/mL of pancreatin, respectively (Table 4).

**Table 4:** Kinetic data for hydrolysis of carvacrol codrug 4 at 37°C.^a^

Chemical hydrolysis		t_½_ (h)	k_obs_ (h^-1^)
**pH 1.3**		75.38 (± 2.94)	0.009 (± 0.001)
**pH 5.0**		62.08 (± 2.11)	0.11 (± 0.01)
**pH 7.4**		22.9 (± 0.6)	0.030 (± 0.001)
**Enzymatic hydrolysis**			
**SGF** ^b^	pepsin 10 mg/mL	74.09 (± 3.19)	0.009 (± 0.001)
	pepsin 40 mg/mL	5.79 (± 0.16)	0.119 (± 0.003)
**SIF** ^b^	pancreatin 10 mg/mL	5.51 (± 0.13)	0.126 (± 0.003)
	pancreatin 40 mg/mL	0.84 (± 0.03)	0.83 (± 0.02)
**Human plasma**		0.50 (± 0.01)	1.38 (± 0.03)
**Rat plasma**		**0.50 (± 0.02)**	1.38 (± 0.05)

^a^Values are means of three experiments, standard deviation is given in parentheses.

^b^Abbreviations: SGF, simulated gastric fluid; SIF, simulated intestinal fluid.

These data evidenced that codrug **4** resulted 13-fold more stable at pH 1.3 respect to pH 7.4 in the presence of pepsin and pancreatin (10 mg/mL), suggesting that the hydrolysis of ester linkage is preferred at physiological pH (Table 4). Codrug **4** showed the same rate of hydrolysis in 80% human and rat plasma (t_½_ = 0.50 h) suggesting that both plasma may have more selective enzymes for the hydrolysis of ester bond (Table 4). Taken together, these results point out that codrug **4** is quite stable to potentially cross unmodified the acidic environment of the stomach, to be absorbed intact from the intestine after oral administration, and able to release carvacrol and Ac-Cys(Allyl)-OH after enzymatic hydrolysis.

Depending on the phospholipid type, PAMPA can mimic different adsorption/permeation environments. For predicting the ability of carvacrol codrug **4** to diffuse through the gastrointestinal tract, lecithin in dodecane has been used for g.i. permeation studies (PAMPA-GI) (Table 5). To carefully reproduce the g.i. tract, the permeability of codrug **4** was determined at pH 5.0, 6.5, and 7.4. Since our carvacrol codrug **4** has a low solubility in the conventional solution used for the PAMPA, permeability tests were performed in the presence of cosolvents (2% of Tween 80 and 10% of MeOH). The employed cosolvents did not change the permeability of the phospholipid layer at the investigated concentrations, as previously reported [37]. After 18 h of incubation, codrug **4** showed good permeability through PAMPA-GI membrane (*P*_*e*_ > 1 x 10^-6^ cm/s) at each value of pH, thus suggesting a good absorption along g.i. tract.

**Table 5:** PAMPA-GI permeability of carvacrol codrug 4 after 16 h of incubation.

PAMPA-GI permeability *P*_*e*_ (10^-6^ cm s^-1^)		
pH 7.4^a^	pH 6.5^a^	pH 5.0^a^
2.34	17	8.6

^a^ pH of both donor and acceptor compartment.

## Conclusion

In summary, we synthesized ten carvacrol codrugs with the aim of developing innovative antimicrobial compounds. However, Ac-Cys(Allyl)-CAR (**4**) resulted the most active compound against *S*. *aureus*, *S*. *epidermidis*, and *E*. *coli* with MIC values of 2.5 mg/mL and endowed with low toxicity. Even though antibacterial activity of Ac-Cys(Allyl)-CAR is not particularly good, pharmacokinetic data evidenced a good stability at pH 1.3 respect to pH 7.4 in the presence of pepsin and pancreatin, suggesting that, after absorption it might be able to release carvacrol and Ac-Cys(Allyl)-OH due to the enzymatic activity.

Further experiments will be necessary to evaluate the *in vivo* antimicrobial activity of Ac-Cys(Allyl)-CAR and assess the real hydrolysis of the ester bond that would guarantee the release of the free hydroxyl group of carvacrol able to form hydrogen bonds and act as a trans-membrane carrier of monovalent cations through the bacterial cytoplasmatic membrane.

In conclusion, new approaches like codrug strategy applied to natural products (essential oils), could be useful in the antimicrobial plan to overcome the issue of the antibiotic resistance.
